# Prevalence of lumbar spondylosis and its association with low back pain among community-dwelling Japanese women

**DOI:** 10.1186/s12891-016-1343-x

**Published:** 2016-12-01

**Authors:** Ritsu Tsujimoto, Yasuyo Abe, Kazuhiko Arima, Takayuki Nishimura, Masato Tomita, Akihiko Yonekura, Takashi Miyamoto, Shohei Matsubayashi, Natsumi Tanaka, Kiyoshi Aoyagi, Makoto Osaki

**Affiliations:** 1 Department of Orthopaedic Surgery, Nagasaki University Graduate School of Biomedical Sciences, 1-7-1 Sakamoto, Nagasaki, 852-8501 Japan; 2 Department of Public Health, Nagasaki University Graduate School of Biomedical Sciences, 1-12-4 Sakamoto, Nagasaki, 852-8523 Japan

**Keywords:** Lumbar spondylosis, Epidemiology, Community-based study, Low back pain

## Abstract

**Background:**

Lumbar spondylosis is more prevalent among the middle-aged and elderly, but few population-based studies have been conducted, especially in Japan. The purpose of this study was to explore the prevalence of lumbar spondylosis and its associations with low back pain among community-dwelling Japanese women.

**Methods:**

Lateral radiographs of the lumbar spine were obtained from 490 Japanese women ≥ 40 years old, and scored for lumbar spondylosis using the Kellgren-Lawrence (KL) grade at lumbar intervertebral level from L1/2 to L5/S1. Height and weight were measured, and body mass index (BMI) was calculated. Low back pain in subjects was assessed using a self-administered questionnaire. Stiffness index (bone mass) was measured at the calcaneal bone using quantitative ultrasound.

**Results:**

Prevalence of radiographic lumbar spondylosis for KL ≥ 2, KL ≥ 3 and low back pain were 76.7%, 38.8% and 20.0%, respectively. Age was positively associated with radiographic lumbar spondylosis (KL = 2, KL ≥ 3) and low back pain. Greater BMI was associated with lumbar spondylosis with KL = 2, but not with KL ≥ 3. Stiffness index was associated with neither radiographic lumbar spondylosis nor low back pain. Multiple logistic regression analysis identified radiographic lumbar spondylosis (KL ≥ 3) at L3/4, L4/5 and L5/S1 was associated with low back pain, independent of age, BMI and stiffness index.

**Conclusion:**

Severe lumbar spondylosis at the middle or lower level may contribute to low back pain.

## Background

Lumbar spondylosis is characterized by disc degeneration and osteophytosis, and is more prevalent among the middle-aged and elderly [1–9]. Since lumbar spondylosis causes low back pain [1, 3, 7, 9–11], it is important to clarify the prevalence, elucidate associated factors, and identify methods to prevent the disease. Although this disorder has been widely studied in clinical settings, few population-based studies have been conducted, especially in Japan [1–20].

Previous studies have shown that the prevalence of lumbar spondylosis ranges from 38% to 85% [1–7, 9, 10, 12–16]. Yoshimura et al. reported that severe lumbar degenerative disease is more common in the United Kingdom than in Japan, possibly due to ethnic differences [13]. Previous studies have shown that lumbar spondylosis is associated with age [1–10, 12, 16], obesity [3, 4, 7, 14] and bone mass [20–22]. On the other hand, some studies have found no association with obesity [5, 10]. Mericonda et al. [18] found a significant positive correlation between ultrasonographic parameters at the calcaneal bone and scores on a degenerative scale that primarily reflect intervertebral disc degeneration only in men, suggesting that associations may differ between sexes. Several studies have found an association between lumbar spondylosis and low back pain [1, 3, 7, 9, 10], but others have not [15, 16, 19]. Whether lumbar spondylosis causes low back pain remains controversial.

The prevalence of lumbar spondylosis and associations with low back pain have yet to be fully elucidated. The purpose of this study was to explore the prevalence of lumbar spondylosis and its associations with low back pain among community-dwelling Japanese women.

## Methods

### Study design and participants

This study was conducted as part of a survey of age-related health status in a Japanese community (the Oshima Health Study). All women ≥ 40 years old in Oshima, a town in Nagasaki Prefecture in Japan, were invited to participate. Oshima has a population of approximately 5,800 (2,850 men, 2,950 women), including approximately 2,000 women ≥ 40 years. Despite having a shipyard in the town, Oshima is primarily a rural area. The examination of each subject was performed at the Oshima Health Center between 2001 and 2003. A total of 573 women (approximately 30% of eligible women) participated in the study. All participants were non-institutionalized, lived independently, and provided written informed consent before examinations. All study protocols were approved by the Ohshima local ethics committee.

### Measurements

Height (in meters) and weight (in kilograms) were measured with the subject in light clothing and without shoes, and body mass index (BMI) was calculated as weight/height^2^. Subjects were asked if they had low back pain on most days during the previous one month using a self-administered questionnaire (yes/no). Information on current smoking and alcohol drinking was also obtained (yes/no). Stiffness index (bone mass) was also measured at the calcaneal bone using quantitative ultrasound (QUS). Broadband ultrasound attenuation (BUA: dB/MHz) and the speed of sound (SOS: m/s) were measured with an Achilles ultrasound bone densitometer (GE Lunar, Madison, WI). Stiffness index, a function of BUA and SOS, was automatically calculated by using the scanner software [23].

### Spine radiographic assessment

**Fig. 1:**
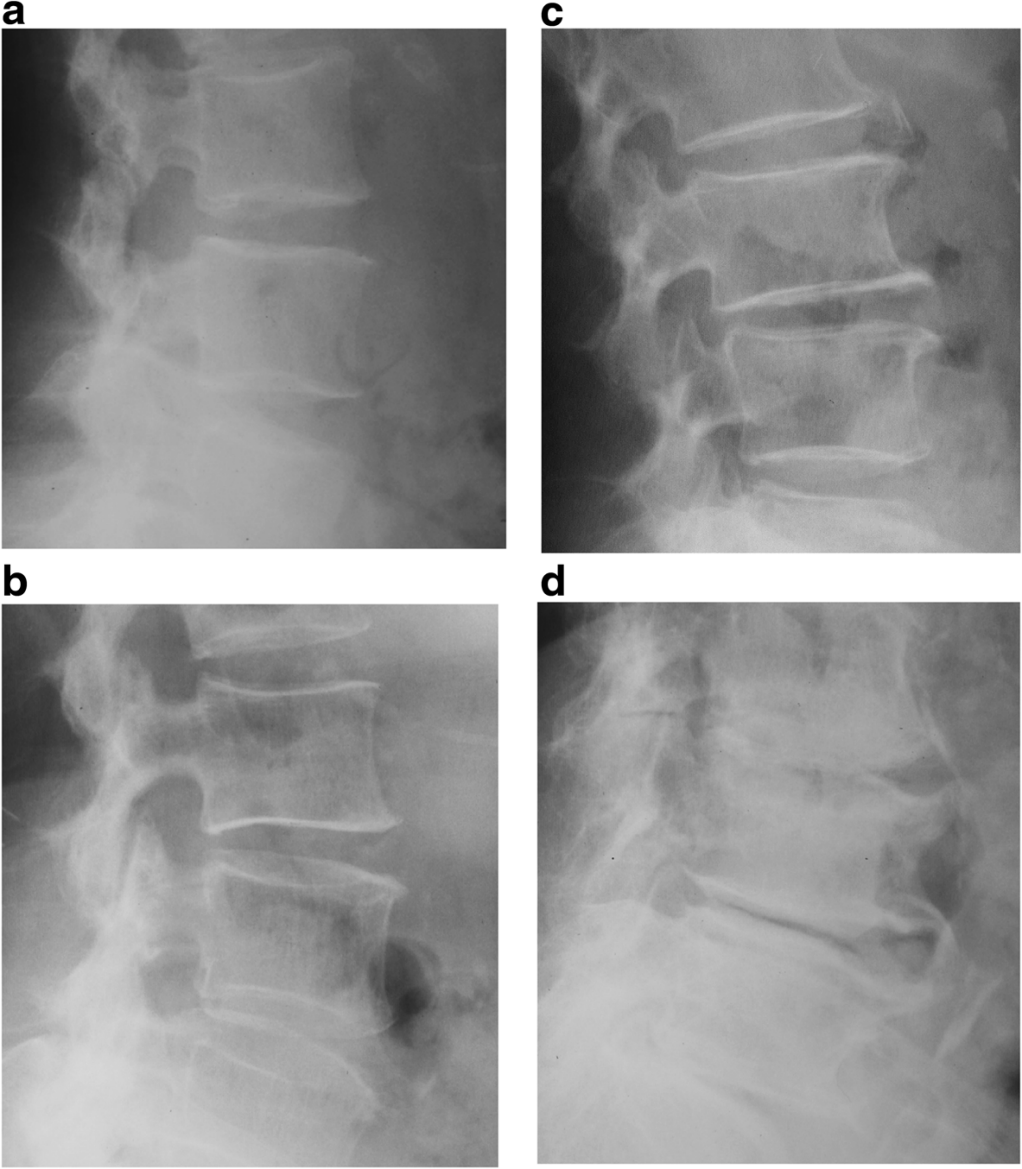
Radiographs were scored for lumbar spondylosis using the Kellgren-Lawrence (KL) grade as follows: KL1, slight osteophytes (**a**); KL2, definite osteophytes (**b**); KL3, disc space narrowing with osteophytes (**c**); KL4, bone sclerosis, disc space narrowing, and large osteophytes (**d**)

Lateral radiographs of the lumbar spine were obtained with the subject lying on their side with knees bent. Radiographs were scored by a single experienced orthopedic surgeon (RT) for lumbar spondylosis using the Kellgren-Lawrence (KL) grade as follows: KL0, normal; KL1, slight osteophytes; KL2, definite osteophytes; KL3, disc space narrowing with osteophytes; KL4, bone sclerosis, disc space narrowing, and large osteophytes [24] (Fig. 1). The present study defined a spine with disc space narrowing with or without osteophytes as KL3. KL grade was determined at the intervertebral level from L1/2 to L5/S1. To evaluate the Intra- observer variability of KL grading, randomly selected radiographs of the lumbar spine were scored by the same reader more than 1 month after the first reading for 50 individuals. Furthermore, 50 other radiographs were scored by two experienced orthopedic surgeons (RT and SM) using the same radiographic atlas for inter-observer variability. Intra- and inter-observer variability was then evaluated by kappa analysis. The radiographic readers (RT and SM) were blind to subject age and other characteristics.

### Statistical analysis

Seventy-seven women for whom radiographs showed poor technical quality and seven women with missing data on low back pain were excluded, leaving 490 women for analysis. The Cochran-Armitage trend test was used to evaluate differences in the prevalence of radiographic lumbar spondylosis or low back pain among age groups. Age-specific means of stiffness index were determined using a general linear modelling method. Logistic regression analysis was used to explore the associations of age, BMI and stiffness index with radiographic lumbar spondylosis (based on the worst level of spondylosis) or low back pain, and the associations of KL grade at each intervertebral level with low back pain, adjusting for age, BMI and stiffness index. Furthermore, we assigned scores as follows: 0, KL = 0–1; 1, KL = 2; and 2, KL ≥ 3. Scores for each level were totaled for each individual (range, 0–10). We conducted logistic regression analysis in order to examine the association between total score for spondylosis at each level and low back pain, adjusting for age, BMI and stiffness index. Results are presented as odds ratios (ORs) with 95% confidence intervals (CIs). Data were analyzed using Statistical Analysis System software package version 9.2 (SAS Institute, Cary, NC).

## Results

Intra- and inter-observer variability in the KL grading of lumbar radiographs were found to be sufficient, with “substantial” kappa scores of 0.78 (95%CI: 0.62-0.94) and 0.63 (95%CI: 0.44-0.83), respectively.

Characteristics of subjects are presented in Table 1. Mean (standard deviation) age, BMI and stiffness index were 64.3 (10.7) years, 23.3 (3.3) kg/m^2^, and 68.8 (16.1), respectively.

**Table 1:** Subject characteristics

No. of subjects	490
Age (years)	64.3 ± 10.7
Height (cm)	150.1 ± 6.53
Weight (kg)	52.4 ± 8.51
BMI (kg/m^2^)	23.3 ± 3.3
Stiffness index	68.8 ± 16.1
Current smoker (%)	4.1
Current drinker (%)	8.8

Data are given as mean ± SD

*BMI* body mass index

Overall prevalence of radiographic lumbar spondylosis for KL ≥ 2, KL ≥ 3 and low back pain were 76.7%, 38.8% and 20.0%, respectively (Table 2). The prevalence of radiographic lumbar spondylosis of KL ≥ 2 (*p* < 0.001), KL ≥ 3 (*p* < 0.001) and low back pain (*p* = 0.006) increased with age. Stiffness index decreased with age (*p* < 0.001). Numbers (%) of subjects with radiographic lumbar spondylosis at each intervertebral level are shown in Table 3. Higher prevalence of radiographic lumbar spondylosis (KL ≥ 2) was seen at L3/4 (50.8%) and L2/3 (48.4%). Higher prevalence of radiographic lumbar spondylosis (KL ≥ 3) was seen at L5/S1 (17.1%) and L4/5 (14.5%).

**Table 2:** Number (%) of subjects with radiographic lumbar spondylosis and low back pain and mean of stiffness index according to age

	*n*	Radiographic lumbar spondylosis						Low back pain		Stiffness index (mean ± SD)
		KL ≥ 2		KL = 2		KL ≥ 3				
Overall	490	376	(76.7)	186	(37.9)	190	(38.8)	98	(20.0)	68.8 ± 16.1
40-49	50	15	(30.0)	12	(24.0)	3	(6.0)	9	(18.0)	86.8 ± 13.4
50-59	111	77	(69.4)	54	(48.7)	23	(20.7)	15	(13.5)	76.9 ± 14.3
60-69	153	121	(79.1)	55	(33.0)	66	(43.1)	26	(17.0)	68.2 ± 12.6
70-79	148	135	(91.2)	53	(35.8)	82	(55.4)	38	(25.7)	60.8 ± 13.0
≥80	28	28	(100)	12	(42.9)	16	(57.1)	10	(35.7)	50.1 ± 12.9
Trend		*p* < 0.001^a^		*p* = 0.98^a^		*p* < 0.001^a^		*p* = 0.006^a^		*p* < 0.001^b^

*n* number, *KL* Kellgren-Lawrence grading; Stiffness index, stiffness index by quantitative ultrasound at calcaneal bone

^a^Cochran-Armitage test

^b^general linear modelling method

**Table 3:** Number and percentage of subjects with radiographic lumbar spondylosis at each intervertebral level

	Overall (*n* = 490)	40-49 (*n* = 50)	50-59 (*n* = 111)	60-69 (*n* = 153)	70-79 (*n* = 148)	≥80 (*n* = 28)
	KL ≥ 2					
L1/2	163 (33.3)	4 (8.0)	17 (15.3)	48 (31.3)	73 (52.7)	21 (75.0)
L2/3	237 (48.4)	6 (12.0)	47 (42.3)	75 (49.0)	88 (59.5)	21 (75.0)
L3/4	249 (50.8)	10 (20.0)	50 (45.0)	82 (53.6)	86 (58.1)	21 (75.0)
L4/5	200 (40.8)	7 (14.0)	31 (27.9)	72 (47.1)	76 (51.4)	14 (50.0)
L5/S1	157 (32.0)	4 (8.0)	21 (18.9)	37 (24.1)	70 (47.2)	15 (53.6)
	KL ≥ 3					
L1/2	48 (9.8)	1 (2.0)	3 (2.7)	12 (7.8)	25 (16.9)	7 (25.0)
L2/3	47 (9.6)	0 (0)	3 (2.7)	15 (9.8)	27 (18.2)	2 (7.1)
L3/4	39 (8.0)	1 (2.0)	2 (1.8)	11 (7.2)	19 (12.8)	6 (21.4)
L4/5	71 (14.5)	0 (0)	7 (6.3)	28 (18.3)	29 (19.6)	7 (25.0)
L5/S1	84 (17.1)	2 (4.0)	13 (11.7)	28 (18.3)	36 (24.3)	5 (17.9)

*n* number, *KL* Kellgren-Lawrence grading

Age was positively associated with radiographic lumbar spondylosis (KL = 2, KL ≥ 3; with worst level selected) and low back pain (Table 4). The OR with a 10-year increase in age was 2.37 in KL = 2, 2.37 in KL ≥ 3 and 1.34 in low back pain. Greater BMI was associated with lumbar spondylosis with KL = 2, but not with KL ≥ 3. The OR with a 1-SD (3.3-kg/m^2^) increase in BMI was 1.59 in KL = 2. BMI was not associated with KL = 3, relative to KL = 0–2 (data not shown). Stiffness index was associated with neither radiographic lumbar spondylosis nor low back pain.

**Table 4:** Associations of age, BMI, and stiffness index with radiographic lumbar spondylosis and low back pain

		Radiographic lumbar spondylosis		Low back pain
	Unit	KL = 2	KL ≥ 3	
		OR (95% CI)	OR (95% CI)	OR (95% CI)
Age (years)	10-year increase	2.37 (1.68-3.16)†	2.37 (1.76-2.92)†	1.34 (1.00-1.71)*
BMI (kg/m^2^)	One SD (3.3) increase	1.59 (1.17-2.11) *	1.10 (0.89-1.32)	1.00 (0.79-1.25)
Stiffness index	One SD (16.1) increase	1.17 (0.81-1.51)	1.00 (0.72-1.19)	0.85 (0.65-1.17)

Radiographic spondylosis was determined at the worst (most severe) level among L1/2–L5/S1

Odds ratios were calculated by logistic regression analysis after adjustment for other variables

*BMI* body mass index, *KL* Kellgren-Lawrence grading, Stiffness index, stiffness index by quantitative ultrasound at calcaneal bone, *OR* odds ratio, *Cl* confidence interval

* *p* < 0.05; † *p* < 0.01

Through multiple logistic regression analysis, Table 5 shows the association of KL grade (KL = 2 and KL ≥ 3) at each intervertebral level with low back pain relative to KL 0 or 1, adjusting for age, BMI and stiffness index. Radiographic lumbar spondylosis (KL ≥ 3) at L3/4, L4/5 or L5/S1 was associated with low back pain, independent of other covariates. We repeated the analysis for the association of KL ≥ 2 with low back pain relative to KL < 2, and of KL ≥ 3 relative to KL < 3. Similar results were obtained; lumbar spondylosis (KL ≥ 3) at L3/4, L4/5 or L5/S1 was associated with low back pain, but lumbar spondylosis (KL ≥ 2) at each level was not, except L5/S1. We conducted logistic regression analysis in order to examine the association between total spondylosis score at each level and low back pain, adjusting for age, BMI and stiffness index. A 1-point increase in spondylosis score was significantly associated with an increased risk of low back pain (OR: 1.2, 95%CI: 1.1-1.3) (data not shown).

**Table 5:** Association of Kellgren-Lawrence (KL) grade (KL = 2, KL ≥ 2 and KL ≥ 3 relative to KL < 2, and KL ≥ 3 relative to KL < 3) at each intervertebral level with low back pain

		L1/2	L2/3	L3/4	L4/5	L5/S1
	reference	OR (95% CI)	OR (95% CI)	OR (95% CI)	OR (95% CI)	OR (95% CI)
KL = 2	KL < 2	1.16	0.87	0.90	1.19	1.55
		(0.66-2.02)	(0.52-1.45)	(0.55-1.48)	(0.68-2.05)	(0.82-2.91)
KL ≥ 2	KL < 2	1.31	1.03	1.07	1.57	1.77*
		(0.80-2.15)	(0.64-1.65)	(0.67-1.70)	(0.99-2.50)	(1.09-2.86)
KL ≥ 3	KL < 2	1.93	1.91	2.57*	2.49*	1.88*
		(0.93-3.99)	(0.92-3.97)	(1.19-5.55)	(1.35-4.56)	(1.06-3.35)
KL ≥ 3	KL < 3	1.66	1.86	2.54*	2.19*	1.76*
		(0.84-3.28)	(0.96-3.62)	(1.26-5.14)	(1.25-3.84)	(1.02-3.03)

Odds ratios were calculated by logistic regression analysis compared with subjects with KL grade 0 or 1 after adjustment for age, body mass index, and stiffness index at calcaneal bone

*OR* odds ratio, *CI* confidence interval

*p < 0.05

## Discussion

We showed that the overall prevalence of radiographic lumbar spondylosis of KL ≥ 2 or KL ≥ 3 and low back pain was 76.7%, 38.8% and 20.0%, respectively, among community-dwelling Japanese women ≥ 40 years old. Previous studies have reported the prevalence as between 38 and 85% [1–7, 9, 10, 12–16]. These differences may be due to age distribution, sex, ethnicity and definition of lumbar spondylosis. Yoshimura et al. [13] reported a difference in the prevalence of lumbar spondylosis between the United Kingdom and Japan.

Muraki et al. [7] showed that in women ≥ 60 years, the prevalence of radiographic lumbar spondylosis with KL ≥ 2, KL ≥ 3 and low back pain were 70.7%, 52.1% and 31.2%, respectively, in a large-scale population study in Japan. Those results were slightly higher than our own. When we selected women ≥ 60 years old as subjects, prevalence of radiographic spondylosis with KL ≥ 2, KL ≥ 3 and low back pain was 86.3% (284/329), 50.2% (165/329) and 22.5% (74/329), respectively, similar to the results above.

Several studies have shown older age to be associated with lumbar spondylosis [1–10, 12, 16] We also showed that older age was associated with lumbar spondylosis of KL = 2 and KL ≥ 3.

In our study, greater BMI was associated with lumbar spondylosis with KL = 2, but not with lumbar spondylosis with KL ≥ 3, which suggests that greater BMI may be associated with moderate lumbar spondylosis. Some studies have reported that obesity is related to lumbar spondylosis [3, 4, 7, 14], but others have not [5, 10]. Yoshimura et al. [13] reported that obesity was related to lumbar spondylosis in the United Kingdom, but not in Japan. According to van Saase et al. [17], the relationship between obesity and lumbar spondylosis differed between sexes. Obesity was related to lumbar spondylosis with KL ≥ 2 in both sexes. On the other hand, obesity correlated negatively with lumbar spondylosis of KL ≥ 3 only in women, not in men. The relationship between obesity and lumbar spondylosis may differ by severity, ethnicity or sex.

In our study, stiffness index from QUS was not associated with lumbar spondylosis. Mariconda et al. [18] studied whether QUS, a radiation-free, easy-to-operate, inexpensive and rapid technique, might prove useful in predicting the extent of degenerative changes in the lumbar spine, showing that stiffness index was positively associated with lumbar disc degeneration in men, but not in women. Furthermore, bone mineral density (BMD) by dual-energy X-ray absorptiometry (DEXA) was reported to be positively associated with lumbar spondylosis or disc degeneration [20–22]. Assessment of the association of BMD with lumbar spondylosis or disc degeneration must be made with caution because of differences in measurement methods (QUS or DEXA) and measurement sites (heel, lumbar or femoral neck).

We showed that a higher prevalence of radiographic lumbar spondylosis (KL ≥ 2) was seen at L3/4 and L2/3, and that a higher prevalence of radiographic lumbar spondylosis (KL ≥ 3) was seen at L5/S1 and L4/5. O’Neill et al. [4] reported that osteophytes occurred most frequently at L2/3 in women. Kramer [6] reported that osteophytes and disc space narrowing were most prevalent at L4/5 in women. Teraguchi et al. [14] reported that the prevalence of disc degeneration in the lumbar spine using magnetic resonance imaging (MRI) was highest at L4/5 in women. These differences may be due to differences in ethnicity, the definition of lumbar spondylosis or the assessment method applied (radiograph or MRI).

The association between lumbar spondylosis and low back pain is controversial. Some studies have reported an association [1, 3, 7, 9–11], but others have not [15, 16, 19]. In our study, lumbar spondylosis (KL ≥ 3) at the middle or lower level was associated with low back pain. On the other hand, the majority of subjects with lumbar spondylosis (KL ≥ 2: 76.7%; KL ≥ 3: 38.8%) did not show low back pain (20.0%) (Table 2). These findings were somewhat conflicting. Furthermore, no standardized methods have been devised for investigating the relationship between lumbar spondylosis and low back pain; different thresholds have been used, such as worst level [1, 7, 10, 11, 15, 17, 19], number of degenerated discs [1, 3, 16, 19], average level [9, 16, 19], and single spinal level [3, 7]. Such differences would inevitably contribute to the variability of findings between studies.

This study has several limitations that must be considered. First, since this study used a cross-sectional design, our results do not necessarily show a causal relationship. Second, data on spinal canal stenosis, degeneration at facet joints, or other spinal disorders or psychological distress causing low back pain were not available in our study. Third, we did not collect data on pain severity. Further study is needed to clarify the severity of low back pain using a visual analogue or numeric pain rating scale to assess the relationship between severity of degenerative changes and severity of low back pain. The KL grade does not discriminate according to the degree of disc space narrowing. Caution is thus needed when interpreting the results. Fourth, a relatively high proportion of women (13.4%: 77/573) was excluded from analysis because of the poor technical quality of radiographs, which may have led to selection bias. Fifth, because this study only included women, our findings may not be generalizable to men.

## Conclusions

Lumbar spondylosis (KL ≥ 3) at L3/4, L4/5 or L5/S1 was associated with low back pain among community-dwelling Japanese women. Severe lumbar spondylosis at the middle or lower level may contribute to low back pain.

## Abbreviations

BMD: Bone mineral density
BMI: Body mass index
CIs: Confidence intervals
DEXA: Dual-energy X-ray absorptiometry
KL: Kellgren-Lawrence
MRI: Magnetic resonance imaging
ORs: Odds ratios
QUS: Quantitative ultrasound
